# How Can We Control the Prevalence of Tuberculosis among Cattle?

**Published:** 1894-10

**Authors:** M. R. Trumbower


### HOW CAN WE CONTROL THE PREVALENCE OF TUBERCULOSIS AMONG CATTLE?*

By M. R. Trumbower, D. V. S.

Addressing you as a representative of a great Western State, one noted for its dairy and breeding interests, and in which no action has yet been taken concerning the control of this disease, I may appear as an obstructionist, rather than an active sanitarian, to those of you who have studied this subject longer than I have, and who have been engaged in practical work for its control.

The fatality among cattle from this disease is not so great as to create a general demand for legal measures to protect breeders and dairymen against losses. The demand is made as a public health measure.

In whatever I may say to you here to-day, I do not wish to be understood that I am opposed to destroying the tuberculous cattle wherever they may be found, but I do believe that the danger to human life, by eating the meat or drinking the milk from tuberculous cattle, is very greatly exaggerated by many of our medical brethren.

In the consideration of this subject we must lay sentiment aside, attractive as it may seem, and study it so that we may arrive at conclusions that are practical and capable of being carried into effect, without becoming abortive in our hands.

While I appreciate the need of aggressive work, I feel that we of the Western States are far from being ready or able to enforce any specially restrictive measures at the present time. Our people are not educated in this matter. Some of our farm journals are publishing reports of the work done by a few of the Eastern States, with comments upon it that are very unfavorable, and, instead of being an aid, are detrimental to correct information.

Our dairymen are suspicious of imposition, and jealous of their rights, and, if we attempt at the present time to enforce active measures, involving the business of the dairymen in the destruction of a part of their herds, before they become aware of the justice of such proceedings, we will succeed only in making a miserable

*Read at a meeting of the United States Veterinary Medical Association, held in Philadelphia, Pa., September 18th–20th, 1894.

failure, and endanger all practical efforts for the next generation.

Even most of our physicians and public health officials are not sufficiently well informed to endorse the actions that will be necessary to secure effective results.

In the medical profession this matter of sanitary protection against tuberculosis infection is in its infancy; by many of them the dangers are depicted in a horrifying manner, and by others they are almost entirely ignored.

Until the medical profession obtains some recognition in its endeavor to protect human life, what have we to hope for? In their efforts, human lives are at stake alone, regardless of financial values. In our efforts, financial values are predominant in the minds of those directly and primarily affected.

Our dairy interests are very great. The dairy products of Illinois in 1892 amounted to the value of $75,691,765; in 1893, $90,000,000 in round numbers. Before we antagonize this great industry in any way that may seem arbitrary, we should count the cost. Invested capital recognizes no sentiment, nor will it tolerate any apparent infringement upon its rights.

While this agitation regarding the prevalence of tuberculosis among cattle is a legitimate and proper one, we should be guarded in our assertions, and confine ourselves to statements of fact. Some there are who dwell upon the great danger resulting to the human family from the consumption of meat and milk, a danger that has been exaggerated out of all proportion, and, if continued, will result in the closing of all foreign markets against the beef and dairy products of this country, for it will lead foreign nations to believe that all our herds are largely tuberculous. We ought to be just toward ourselves, and avoid magnifying a fact that is sufficiently serious to be worthy of our attention when viewed in the light of truth.

Why lay so much blame upon milk from cows, by the wild presumption that many thousand infants die annually from intestinal tuberculosis, the result of milk infection, when it is acknowledged that seven per cent. of the human family is consumptive?

Does it appear right?

How many cases are recorded when cows' milk was the known cause of such fatality? Very few.

Does it not appear to you that we are attempting to make a scapegoat of the useful and almost innocent cow? Again I say, we are not yet ready in the West to adopt radical measures for the suppression of a disease that does not prevail to any alarming extent among our herd.

I am well satisfied that we in Illinois do not have the percentage of tuberculous animals that you have in Massachusetts, or any other of the Eastern States. Our cattle are kept under different, better, and more natural conditions Their lives are more active, they are not so closely housed, there is far less exposure to contamination, and, above all, are not inbred as much as many dairy herds of the older States. The farmer does not keep the same family of cows on his farm for several successive generations, and then distribute them among his children as part of their birthright.

The majority of our cows are used only one or two years in the dairy, and then they are fattened for beef, while yet young, and desirable beef cattle. The most of our beef cattle are sold and slaughtered before they have gained maturity; hence we rarely find any evidence of tuberculosis among them. Therefore, I believe, we have a great deal less of this disease than is found in the older States.

If we undertake the eradication of tuberculosis from our breeding and dairy herds, we assume an endless task. When we destroy one per cent. or forty per cent. of our dairy cattle, they will have to be replaced by an equal number, which, in turn, may be infected with tuberculosis. We will have to destroy or abandon many cattle barns, such as cannot be thoroughly disinfected.

Consumptive attendants may be a source of infection of our herds. The presence of tuberculosis in swine, and other domestic animals, is not a source of danger.

I see only one way by which we of the West can make any progress towards lessening the prevalence of this malady, and that is, to provide by special enactment laws for the inspection of all cattle that are used for supplying milk to cities, towns and villages. This would require inspectors for each district, who should be appointed by some central power, and no one should be allowed to sell milk coming from uncertified cattle. This action necessarily invokes a large appropriation for compensation of inspectors, and other clerical aid. So long as any community is opposed to such enactment, its representatives in the legislative body will not vote for it. A rural representative will not misrepresent his constituency in such a measure.

Cities, by ordinances, may require milk inspection, but that does not reach far enough ; dairy inspection they cannot enforce, for most of the dairy herds are owned outside of their jurisdiction.

If the State provides for dairy inspection at public expense, all cities, towns and villages are equally entitled to the benefits. Then, where will we find a sufficient number of competent, faithful, and honest men to fill this requirement? We do not have a sufficient number of veterinarians in the State of Illinois to do this work if all were engaged in it, nor is there a single State that has, with the exception of a few of the New England States.

I have an impression that we of the West can obtain the best results for the present time by using all our energies in disseminating reliable information, and thereby arouse public sentiment that will eventually lead to the desired end. Patience, and persistent effort, judiciously administered, will do more good now than more active measures that would be in great danger of being regarded as arbitrary and unjustifiable by our people.

In the meantime, we in the State of Illinois shall make inspections, as heretofore, of herds that are reported to be tuberculous by their owners, and earnestly advise the prohibition of the sale of milk from such herds, until the affected animals have been killed.

By the terms of the law under which our State Board of Live Stock Commissioners act, they can only cause to be investigated reported existence of disease, hence a thorough search for diseased animals cannot be inaugurated except in tracing out exposures to any reported case.

Again, the peculiar wording of the law regarding compensation to owners of diseased animals is such that no compensation can be made in cases of tuberculosis, since in appraising cattle the amount of appraisement is based upon the fair cash market value for use for beef or for dairy purposes. Such being the situation, the only course left for the Board to pursue upon the discovery of this disease, is to place the diseased animals under quarantine, until the owner agrees to destroy them.

We dare not advocate radical measures just now, and I do not consider it is so urgent and vital a necessity as some of our theorists do.

Now, a word in regard to the tuberculin test. Are we justified in condemning as unfit for human food every carcass that has reacted to this test? If so, do we not destroy a great deal of valuable and productive property needlessly?

I do not believe that the milk or flesh is dangerously infected in an animal that has only a small deposit in the mediastinal gland, or an encysted abscess in the liver; but how can we determine this during the lifetime of the animal? We cannot do so, therefore we have to destroy all that react, or else only those which manifest some physical evidence of the disease on examination. Would not the latter method be practically sufficient and exceedingly more satisfactory to the owner than the former?

Re-examinations would have to be made oftener, but we would avoid the antagonism and enmity of the owner, which would eventually lead to submission on his part to any measure we might subsequently advocate.

If it is advisable to destroy all cattle that react to the tuberculin test, the United States Government will have to take charge of this work in all the States that give their consent to it, and all cattle from other States must be strictly excluded, unless they have undergone the test, and are declared free from the disease. This would virtually establish a dead line between States, and would soon become so irksome as to cause organized opposition and greatly delay, if not prove detrimental to all concerned.

Before leaving this subject, I wish to call your attention to a marked element of danger in the diagnosis of tuberculosis post-mortem.

Many of you doubtless know that aged cows which have been badly kept and whose vital powers are on the decline, very frequently have glandular indurations, caseation, and occasionally calcification may be present, without being tubercular in their nature. Again, we occasionally find actinomycotic growths in the lungs and other organs which simulate in appearance tuberculosis so closely that it becomes very difficult to distinguish the difference. Possibly there are veterinarians who might inadvertently swell the percentage of tuberculosis by mistaking such cases for that disease.

I will now leave this subject with you for your consideration, and if I have succeeded in provoking a discussion upon this difficult problem, "How to Suppress Tuberculosis," I shall feel myself amply rewarded for presenting the practical part of this question in my own way.

